# Imaging the human hippocampus with optically-pumped magnetoencephalography

**DOI:** 10.1016/j.neuroimage.2019.116192

**Published:** 2019-12

**Authors:** Daniel N. Barry, Tim M. Tierney, Niall Holmes, Elena Boto, Gillian Roberts, James Leggett, Richard Bowtell, Matthew J. Brookes, Gareth R. Barnes, Eleanor A. Maguire

**Affiliations:** a Wellcome Centre for Human Neuroimaging, UCL Queen Square Institute of Neurology, University College London, London, WC1N 3AR, UK; b Sir Peter Mansfield Imaging Centre, School of Physics and Astronomy, University of Nottingham, University Park, Nottingham, NG7 2RD, UK

**Keywords:** Optically-pumped magnetometers, Magnetoencephalography, Hippocampus, Source localisation, Scene construction, Imagination

## Abstract

Optically-pumped (OP) magnetometers allow magnetoencephalography (MEG) to be performed while a participant’s head is unconstrained. To fully leverage this new technology, and in particular its capacity for mobility, the activity of deep brain structures which facilitate explorative behaviours such as navigation, must be detectable using OP-MEG. One such crucial brain region is the hippocampus. Here we had three healthy adult participants perform a hippocampal-dependent task – the imagination of novel scene imagery – while being scanned using OP-MEG. A conjunction analysis across these three participants revealed a significant change in theta power in the medial temporal lobe. The peak of this activated cluster was located in the anterior hippocampus. We repeated the experiment with the same participants in a conventional SQUID-MEG scanner and found similar engagement of the medial temporal lobe, also with a peak in the anterior hippocampus. These OP-MEG findings indicate exciting new opportunities for investigating the neural correlates of a range of crucial cognitive functions in naturalistic contexts including spatial navigation, episodic memory and social interactions.

## Highlights

• Optically-pumped (OP) magnetometers are advancing magnetoencephalography (MEG).
• OP-MEG brain scanning can be performed while the head is unconstrained.
• We combined OP-MEG with a hippocampal-dependent task.
• Task-related changes in theta power in the hippocampus were detectable.
• We observed similar hippocampal engagement in the same participants using SQUID-MEG.

## 1 Introduction

The development of optically-pumped (OP) magnetometers represents a significant evolution of magnetoencephalography (MEG) technology (Boto et al., 2018). As OP-MEG does not require cryogenic cooling, the sensors can be placed directly on the scalp, and the resulting proximity to the brain increases the magnitude of the measured OP-MEG signal (Boto et al., 2017; Iivanainen et al., 2017). Another significant advance associated with OP-MEG is the capacity for participant movement. During a conventional Superconducting Quantum Interference Devices (SQUID) MEG or functional magnetic resonance imaging (fMRI) scan, participants must remain still to avoid compromising the localisation of brain activity. OP-MEG sensors, on the other hand, can be mounted on a scanner-cast moulded specifically to the participant’s head, allowing the individual to move freely (Boto et al., 2018) when combined with an innovative approach to nulling the magnetic field surrounding the participant (Holmes et al., 2018).

The potential, therefore, exists to study more complex behaviours in which movement plays an integral part. For example, spatial navigation in a naturalistic setting involves changes in head direction and coordinated body movements. However, to fully leverage the wearability of OP-MEG in such a real-world domain, one must be able to detect signal from brain structures upon which behaviours like navigation depend, such as the hippocampus (Maguire et al., 2006). This brain region is located deep in the medial temporal lobe, and the distance from MEG sensors affects the sensitivity with which hippocampal activity can be detected (Hillebrand and Barnes, 2002). However, simulations of hippocampal activation (Meyer et al., 2017) and the deployment of hippocampal-dependent tasks (Fuentemilla et al., 2014; Garrido et al., 2015; Kaplan et al., 2012) have demonstrated that the reconstruction of hippocampal source activity can be achieved with conventional SQUID-MEG. Recently, OP-MEG has been used to successfully localise and lateralise cortical responses in a verb generation task (Tierney et al., 2018), providing the first evidence of accurate cortical source reconstruction with a cognitive task. Analyses of simulated OP-MEG data suggest that sources deeper in the brain could be reconstructed with an accuracy comparable to a SQUID-MEG system (Boto et al., 2016; Iivanainen et al., 2017). However, to date, there exists no empirical evidence that hippocampal activity can be successfully imaged during the performance of a cognitive task using OP-MEG.

One cognitive task which is strongly associated with hippocampal integrity is the mental construction of scene imagery (Hassabis et al., 2007a). Accordingly, performance of this task in an fMRI context elicits robust and specific engagement of the anterior hippocampus (Dalton et al., 2018; Hassabis et al., 2007b; Zeidman et al., 2015a,b; Zeidman and Maguire, 2016). We recently adapted this task for use in SQUID-MEG, using single word cues to rapidly evoke the mental imagery of scenes. We observed changes in theta power (4–8 Hz) in the anterior hippocampus during scene construction when compared to a low-level baseline task (Barry et al., 2019). Therefore, having developed a paradigm which reliably elicits anterior hippocampal activity using SQUID-MEG, here we deployed the same task using OP-MEG technology to determine if successful localisation of hippocampal activity was also possible using these novel sensors. Given the previous fMRI, SQUID-MEG and simulation findings noted above, we predicted that OP-MEG would be sensitive to task-related modulation of hippocampal theta. As a further sanity check, the participants who took part in the OP-MEG experiment also performed the same task in a conventional SQUID-MEG scanner, where we predicted similar modulation of hippocampal theta.

## 2 Materials and methods

### 2.1 Participants

Three participants took part in both the OP-MEG and SQUID-MEG experiments, one female and two males, aged 27, 42 and 50 respectively, with normal or corrected-to-normal vision and no history of psychiatric or neurological disorders. OP-MEG data collection took place at the University of Nottingham, UK. The research protocol was approved by the University of Nottingham Medical School Research Ethics Committee and written informed consent was obtained from all participants. The SQUID-MEG data collection took place at University College London, UK. Participants gave written informed consent, and the University College London Research Ethics Committee approved the study.

### 2.2 Experimental paradigm

Participants performed the same task during both OP-MEG and SQUID-MEG scans. The task involved the imagination of novel scenes in response to single scene words presented one at a time (e.g. “casino”, “boardroom”). The scene stimuli were utilised in a recent fMRI study (Clark et al., 2018), and were rated as both highly imageable and scene-evoking by at least 70% of an independent sample of participants (Clark et al., 2018). An additional baseline condition involved counting, and these number stimuli were matched to the scene words in terms of the number of letters and syllables.

**Fig. 1:**
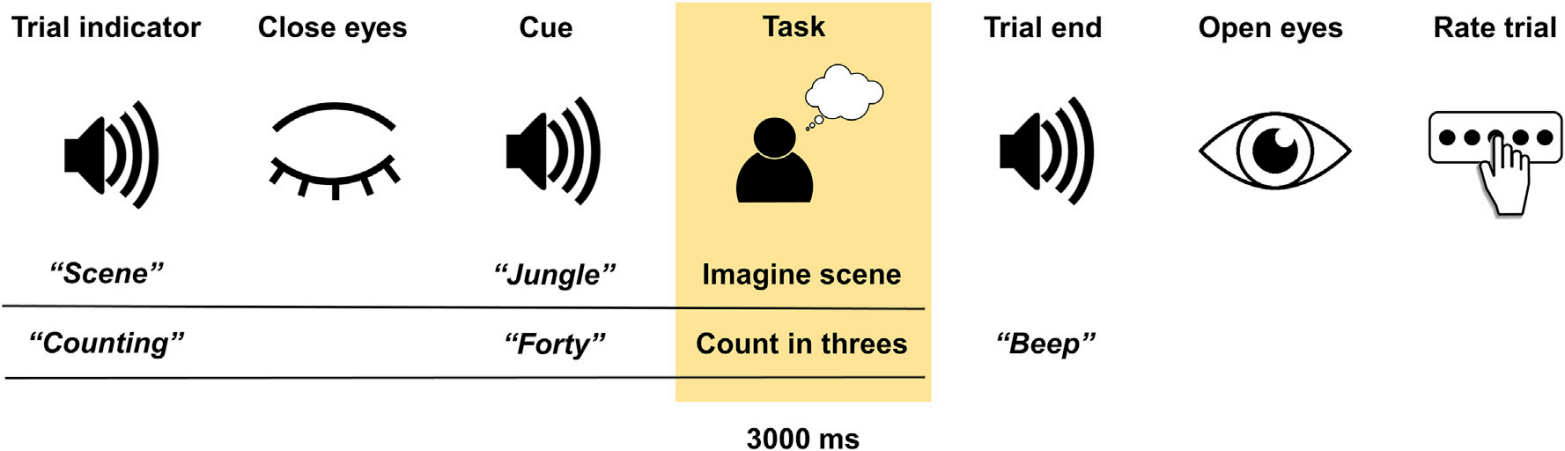
Trial structure. The task period used in the analyses is highlighted.

During scanning, experimental stimuli were delivered aurally via MEG-compatible earbuds using the Cogent toolbox (www.vislab.ucl.ac.uk/cogent.php), running in MATLAB. To prepare participants for each trial type, they first heard either the word “scene” or “counting” (Fig. 1). Participants immediately closed their eyes and waited for an auditory cue which was presented following a jittered delay of between 1300 and 1700 ms. During scene trials, participants constructed a novel, vivid scene in their imagination based on the cue (e.g. “jungle”). Counting trials involved mentally counting in threes from a number cue (e.g. “forty”). The task periods were 3000 ms in duration. Participants then heard a beep, opened their eyes and were presented with a question displayed on the screen in front of them to which they responded using a keypad. They were asked to rate whether or not they were successful in the scene imagination task, and whether or not they paid attention during the counting trials. Following this there was a 1000 ms delay before the next trial. Only trials in which participants affirmed they were successful were subsequently analysed.

In the OP-MEG experiment, participant one completed 45 scene and 45 counting trials, of which 35 scene and 45 counting trials were rated as successful. Participant two completed 75 scene and 75 counting trials, of which 68 scene and 75 counting trials were rated as successful. Seven scene trials and six counting trials were removed from this participant’s data following identification of artefacts. Participant three completed 48 scene and 48 counting trials, of which 45 scene and 48 counting trials were rated as successful. Trial numbers across conditions were equivocated for subsequent analyses, therefore in the OP-MEG datasets 35, 61 and 45 trials in each condition were analysed for participants one, two and three respectively. In the SQUID-MEG experiment, participant one completed 54 scene and 54 counting trials, of which 44 scene and 54 counting trials were rated as successful. Participant two completed 75 scene and 75 counting trials, of which 70 scene and 75 counting trials were rated as successful. Participant three completed 75 scene and 75 counting trials, of which 72 scene and 75 counting trials were rated as successful. Trial numbers across conditions were again equivocated for subsequent analysis, therefore in the SQUID-MEG experiment 44, 70 and 72 trials in each condition were analysed for participants one, two and three respectively.

### 2.3 Data acquisition

#### 2.3.1 OP-MEG

The OP-MEG acquisition system used here was identical to that reported in a previous OP-MEG study which examined neocortical responses during language processing (Tierney et al., 2018). A description of the sensors has also been provided previously (Osborne et al., 2018; Shah and Wakai, 2013). In summary, 28 QuSpin (https://quspin.com/) OP-MEG sensors were available for use. Additionally, motion tracking was performed using an OptiTrack V120 Duo infrared camera system (https://optitrack.com/) in two of the three participants. For the two motion-tracked participants, over the course of the experiment they shifted a median of 23 mm and 12 mm from their initial starting point. The variability (median absolute deviation) of their position was estimated to be 6 mm and 7 mm respectively. Twenty-one sensors that were not transparent to interference from the OptiTrak infra-red illumination were used.

**Fig. 2:**
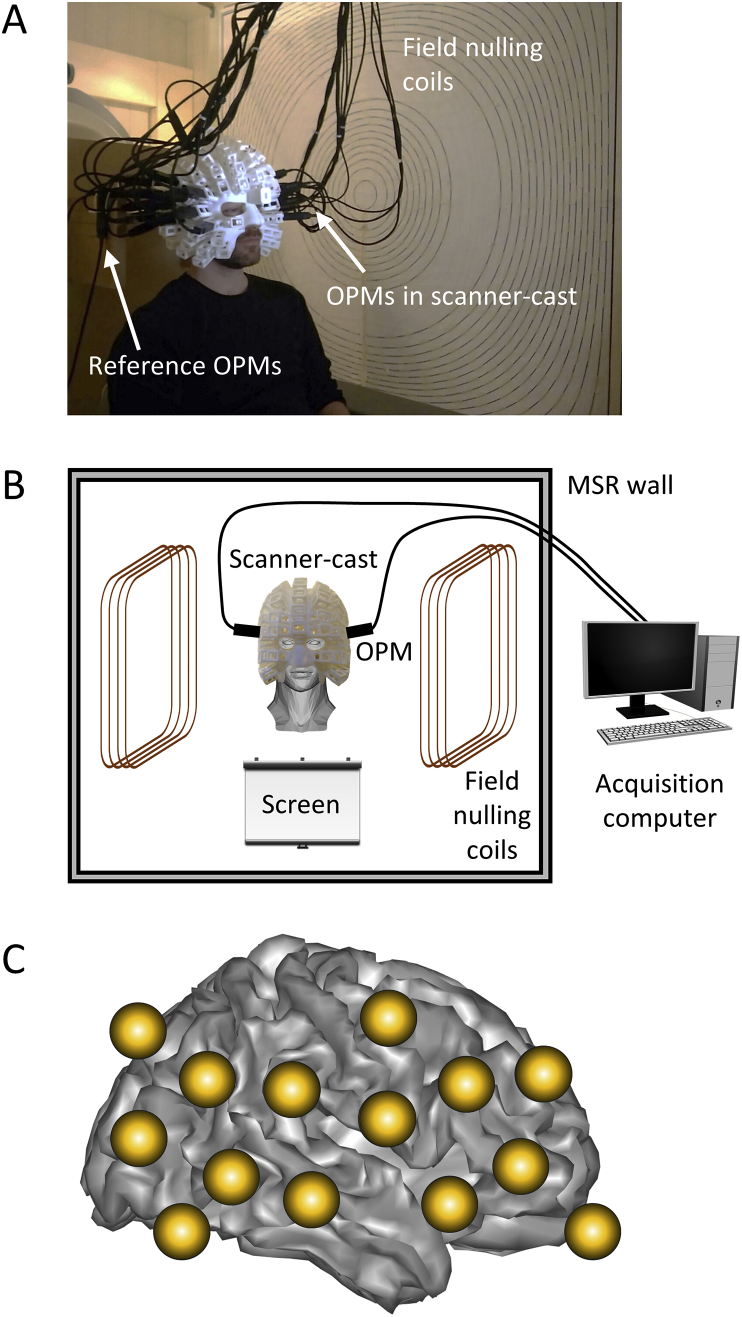
The OP-MEG setup for studying hippocampal engagement during the construction of scene imagery. (A) The participant was seated inside a magnetically shielded room wearing the scanner-cast with OP-MEG sensors inserted into slots. (B) The field nulling coils were placed either side of the participant and conferred motion robustness to the recordings by nulling the field over a 40 × 40 × 40 cm^3^ volume within which head movement was tolerated. (C) The OP-MEG array covered inferior and superior occipital, temporal and frontal areas bilaterally.

The available OP-MEG sensors were attached to the scalp using individualised scanner-casts (Fig. 2A). The scanner-casts were constructed from high bandwidth, low echo time T1-weighted MRI images that allowed accurate reconstruction of the scalp surface (Tierney et al., 2018). Once the scalp surface was reconstructed, a scanner-cast with slots to house the sensors was 3D printed (http://www.chalkstudios.co.uk/). As the scanner-cast was designed from an MRI image, the positions of the sensors relative to the brain were known with a high degree of accuracy, facilitating source reconstruction. A second array of four OP-MEG “reference” sensors were placed in a fixed position directly behind the participant’s head (~10 cm) and were used to model the magnetic interference in the room by regressing their signal from those of the head-mounted sensors. The signal from the reference sensor array (measuring 3 orthogonal components of the Earth’s field as well as 3 spatial gradients) was used to set optimal currents in the field nulling coils to minimise the residual Earth’s magnetic field surrounding the participant’s head (Holmes et al., 2018). The field nulling coils conferred motion robustness on the recordings and allowed the participants to perform the experiment while unconstrained (Boto et al., 2018; Holmes et al., 2018) (Fig. 2B). The dynamic range of the system was set to ± 1.5 nT (although a larger dynamic range of ± 4.5 nT could also have been chosen) with a 0–135 Hz bandwidth (3 dB point) and the recording was digitised using a 16-bit National Instruments digital acquisition system (http://www.ni.com/). The signal was sampled at 1200 Hz following application of an anti-aliasing hardware filter at 500 Hz. The sensors were positioned over inferior and superior occipital, temporal and frontal areas bilaterally (Fig. 2C).

#### 2.3.2 SQUID-MEG

The SQUID-MEG experiment was performed using a CTF Omega whole-head MEG system with 273 functioning second order gradiometers. Data were recorded at a sample rate of 1200 Hz. A left and right preauricular fiducial was utilised in addition to a fiducial on the nasion to aid in source localisation.

### 2.4 Preprocessing

#### 2.4.1 OP-MEG

Data were epoched into 3-s scene imagery and counting periods, baseline corrected, and concatenated across sessions. As OP-MEG sensors are magnetometers, they are susceptible to environmental interference. To mitigate this effect, we constructed synthetic gradiometers (Boto et al., 2017; Fife et al., 1999). We did this by linearly regressing the broadband signal recorded by the reference OP-MEG array from the signal recorded at the scalp OP-MEG array. This was done on a trial-by-trial basis to account for any temporal non-stationarity in the interference.

#### 2.4.2 SQUID-MEG

Data were epoched into 3-s scene imagery and counting periods, baseline corrected, and concatenated across sessions.

### 2.5 Data analysis

#### 2.5.1 OP-MEG source localisation

To identify whether or not OP-MEG could detect the expected signal in the hippocampus, we applied a scalar linearly-constrained, minimum-variance beamformer algorithm implemented in the DAiSS toolbox for SPM (https://github.com/spm/DAiSS). The source orientation was set as the direction of maximal power. We used the Nolte single shell forward model (Nolte, 2003), implemented in SPM12, using the inner-skull boundary derived from the individual T1-weighted MRI. The mapping from sensor to source level (i.e. the beamformer weighting) was estimated using a single covariance window covering the whole trial (0–3000 ms for both scene imagination and counting tasks) in the 4–8 Hz frequency band (5th order bi-directional Butterworth filter). Using these weights, we generated a statistical parametric map (F-statistic) of the power-change in the 4–8 Hz frequency band between the 3000 ms scene imagination task period and the 3000 ms counting baseline period. For comparison across participants, the statistical parametric map was transformed to Montreal Neurological Institute (MNI) space using the nonlinear transformation estimated from the registration of the native T1-weighted MRI to the MNI template sampled on a 3 mm grid. The resulting images were smoothed using a 9 mm Gaussian kernel. In a follow-up analysis, we performed T-contrasts between the two conditions for each participant to ascertain whether observed changes represented an increase or decrease in power.

After localising the task-based modulation of theta power in the source space of each participant, we proceeded to analyse the OP-MEG data at the group level. We performed a conjunction analysis across the three MNI-normalised OP-MEG statistical parametric maps. This analysis allows one to infer a particular brain region’s involvement in a paradigm when multiple participants display similar patterns of activity in this region. The conjunction analysis is based on the maximum p-value method as implemented in SPM12 (Friston et al., 1999, 2005; Worsley and Friston, 2000). We corrected for multiple comparisons across the entire brain volume using False Discovery Rate (FDR) with a conservative threshold of q < 0.005. Localisation was determined based on the MNI coordinates associated with the peak of each separately-identified cluster, in conjunction with the AAL atlas (Tzourio-Mazoyer et al., 2002).

We then sought to quantify the stability of the significant clusters observed in the OP-MEG conjunction analysis through a bootstrapped analysis. For each participant, we resampled their usable trials (with replacement) and generated a new beamformer image of the contrast of interest. We repeated this procedure 100 times per participant. We randomly selected one of the 100 images from each participant and repeated the conjunction analysis on these three images. This procedure was performed 500 times. In each analysis, where a voxel was significant it was assigned a value of one, otherwise it was coded as zero. This yielded 500 binary images of significant voxels, which were summed into one composite image. The value of each voxel was then expressed as a percentage of the total number of conjunctions, to give an indication of how consistently this voxel was significant across bootstrapped analyses.

#### 2.5.2 SQUID-MEG source localisation

The source reconstruction analysis pipeline was the same for SQUID-MEG as the OP-MEG experiment except that analysis was performed in MNI space and coregistration was estimated from the preauricular and nasion fiducials.

## 3 Results

### 3.1 OP-MEG source localisation

**Fig. 3:**
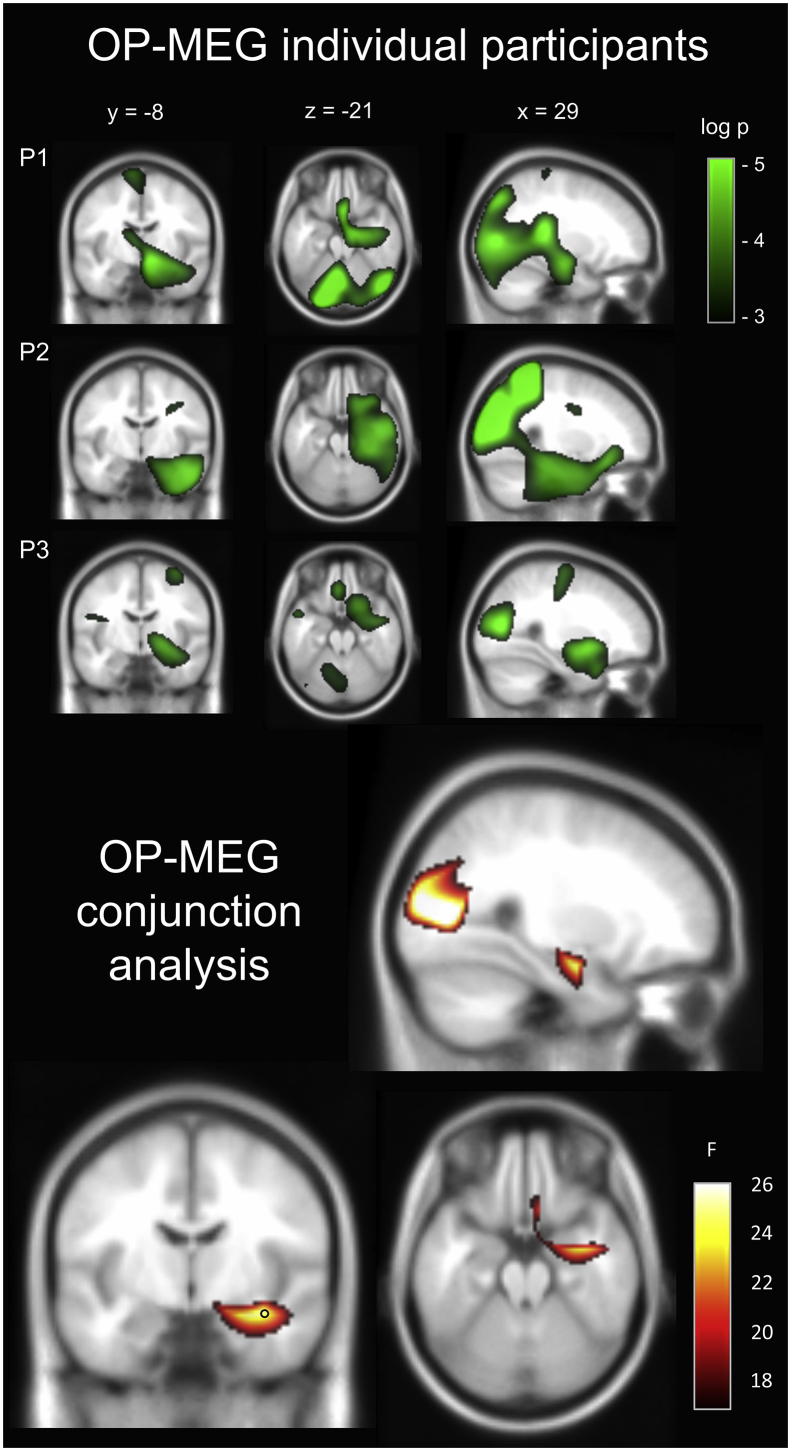
Results of the OP-MEG study. Top panel: Source level theta (4–8 Hz) power changes for each participant during the imagination of novel scenes. P = participant. Images are superimposed on the MNI 152 T1 image. Images are thresholded at a significance level of p < 0.05 (uncorrected for display purposes). Bottom panel: A conjunction analysis of the three participants revealed significant activation of the right anterior hippocampus. The location of peak activity in the hippocampus is indicated with a black circle. Task-based modulation of theta power was also observed in the occipital lobe, with the peak of this large cluster localised to the cuneus. This is not surprising, given the comparison of rich scene imagery with the low level counting baseline task which had no imagery requirement. Previous neuroimaging studies have documented similar engagement of visual cortex during imagery as that evoked by perception of the same content (Albers et al., 2013; Nishimura et al., 2015). Images are FDR thresholded at q < 0.005. This result can also be examined here: https://doi.org/10.6084/m9.figshare.8681483.

Fig. 3 (top panel) displays the changes in theta power during the imagination of novel scenes when contrasted with the baseline counting condition for each individual participant in the OP-MEG experiment. A conjunction analysis across the three participants (bottom panel) revealed a cluster of significant voxels (q < 0.005) in the medial temporal lobe with a peak in the right anterior hippocampus (x = 36, y = −8, z = −16, F statistic = 25.19). This activation extended into the parahippocampal cortex, temporal cortex, amygdala and also the ventromedial prefrontal cortex. A second posterior cluster was observed with a peak in the cuneus (x = 26, y = −76, z = 10, F statistic = 34.95). This cluster also encompassed the calcarine sulcus, mid and superior occipital gyrus bilaterally as well as the right precuneus and superior parietal cortex. A third cluster was observed proximal to this, with a peak in the right paracentral lobule (x = 4, y = −40, z = 74, F statistic = 18.78), extending into the precuneus bilaterally. Subsequent T-contrasts performed on each participant’s data revealed that the power changes almost exclusively represented a theta decrease in the scene imagination condition relative to the baseline task. Specifically, this power decrease was evident in 99.95%, 99.34% and 99.7% of all significant voxels for participant one, two and three respectively. All significant voxels in the group conjunction analysis represented a power decrease at the single participant level.

**Fig. 4:**
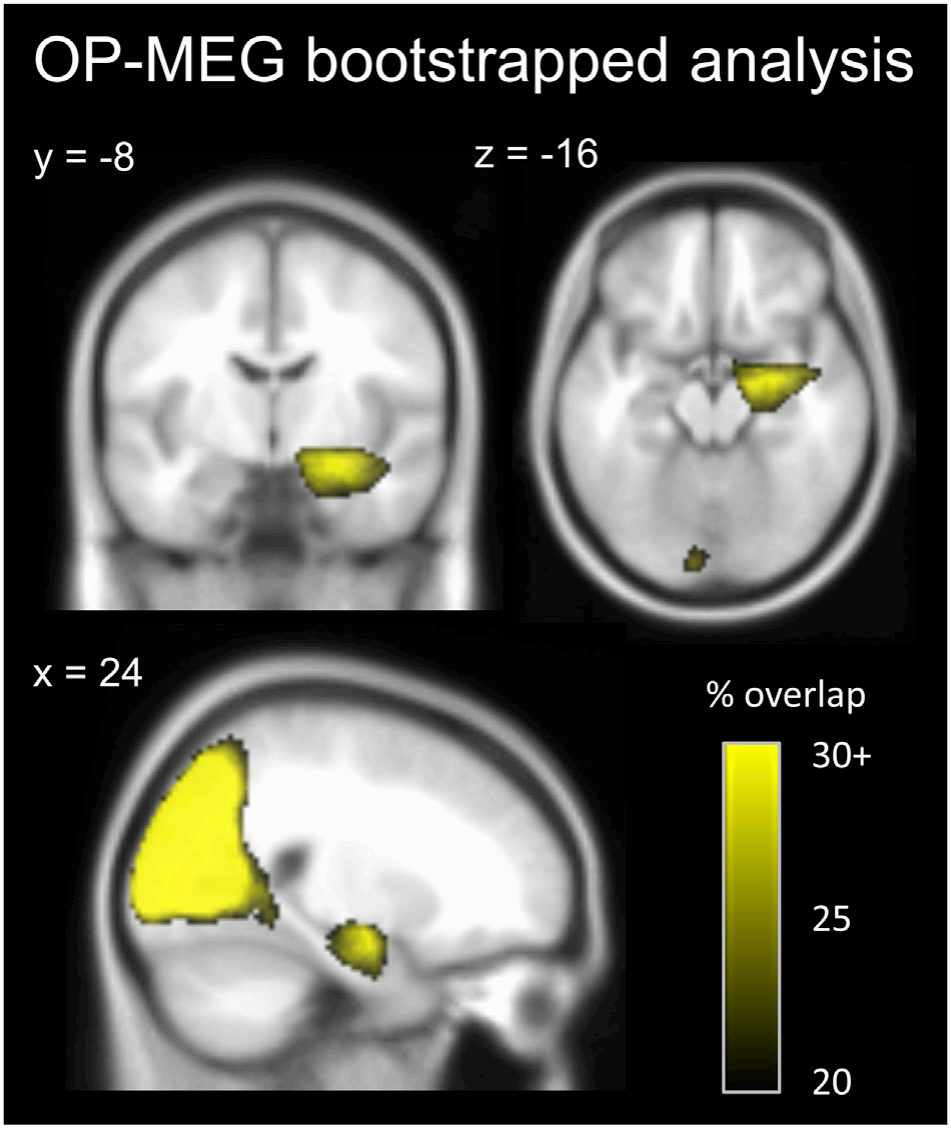
Results of the bootstrapped OP-MEG conjunction analyses. The conjunction analysis was repeated 500 times with sub-sampling of each participant’s trials to give an indication of the stability of the observed significant clusters. Within the medial temporal lobe, the location of the highest concentration of conjunctions where voxels displayed significant activity was in the right anterior hippocampus. Occipital regions which emerged from the original conjunction analysis were also reliably engaged. The map of the percentage of significant conjunctions is superimposed on the MNI 152 T1 image, at an arbitrarily chosen minimum threshold of 20% overlap across all conjunctions.

By bootstrapping the trials of each participant and repeating the conjunction analysis 500 times, we generated an image which characterised the stability of the significant clusters observed. Fig. 4 displays the brain areas which generated the most common engagement across these analyses. The region which displayed the most consistent engagement across these conjunctions in the medial temporal lobe was again the right anterior hippocampus (peak voxel x = 24, y = −8, z = −16). Highly consistent results were also observed in posterior regions, with a peak in the superior occipital gyrus (x = 22, y = −80, z = 16).

### 3.2 SQUID-MEG source localisation

**Fig. 5:**
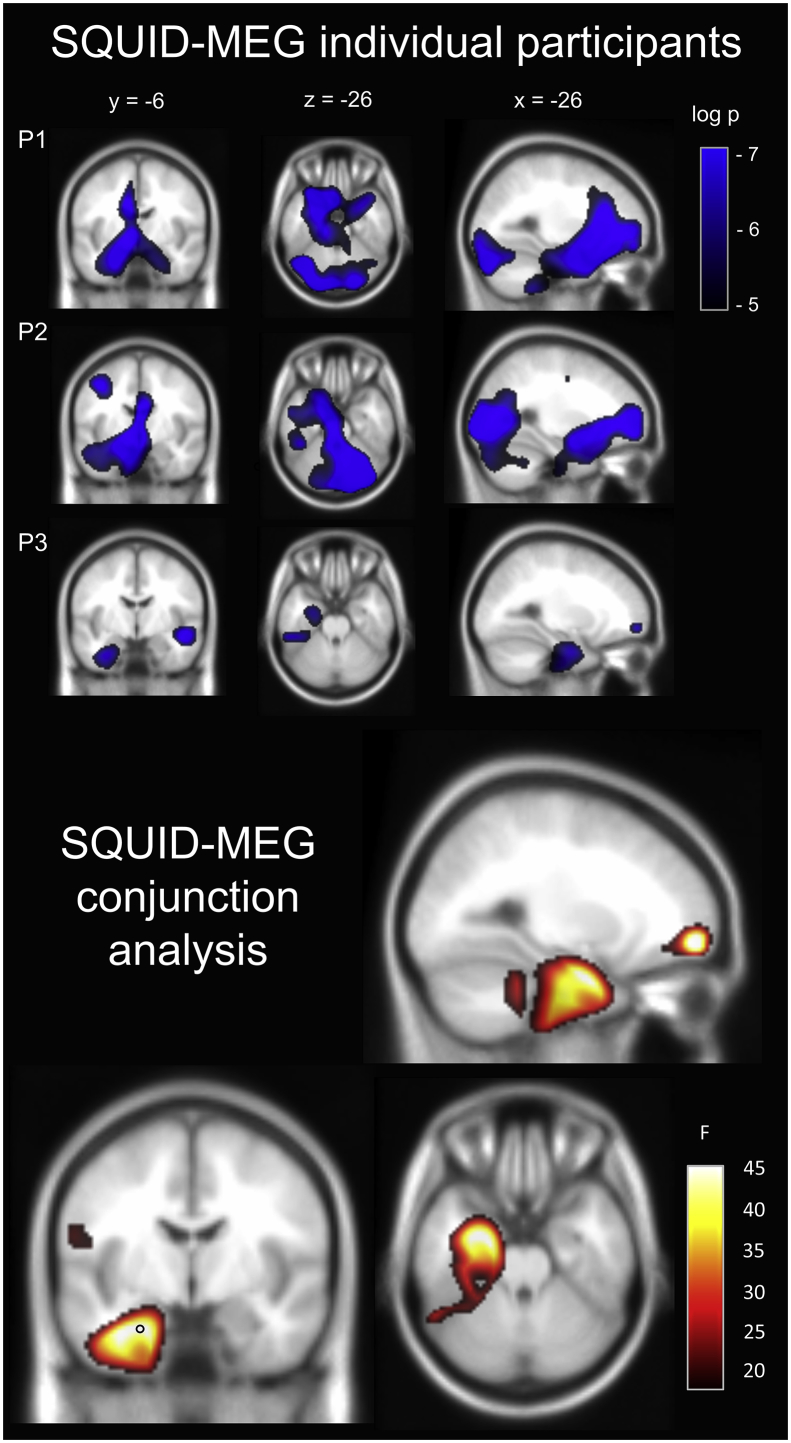
Results of the SQUID-MEG study. Top panel: Source level theta (4–8 Hz) power changes for each participant during the imagination of novel scenes. P = participant. Images are superimposed on the MNI 152 T1 image. Images are thresholded at a significance level of p < 0.007 (uncorrected for display purposes). Bottom panel: A conjunction analysis of the three participants revealed significant activation of the left medial temporal lobe. The peak of this cluster was located in the anterior hippocampus (indicated with a black circle). Task-based modulation of theta power was also observed in the left frontal gyrus. Images are FDR thresholded at q < 0.005. This result can also be examined here: https://doi.org/10.6084/m9.figshare.8681483.

Fig. 5 (top panel) displays the changes in theta power during the imagination of novel scenes when contrasted with the baseline counting condition for each of the individual participants in the SQUID-MEG experiment. A conjunction analysis across the three participants (bottom panel) revealed that the largest cluster of significant voxels (q < 0.005) was centred on the medial temporal lobe with a peak in the left anterior hippocampus (x = −26, y = −6, z = −26, F statistic = 47.73). This activation extended into the fusiform, parahippocampal and temporal cortices, amygdala, occipital gyrus and the cerebellum. A whole-brain peak was observed in a separate cluster in the superior frontal gyrus (x = −26, y = 56, z = −8, F statistic = 54.18).

## 4 Discussion

In this study, we deployed novel OP-MEG technology to measure brain activity during the performance of a validated hippocampal-dependent task. We demonstrated that, even when participants’ movements were unconstrained, task-related modulation of theta power in the medial temporal lobe was observable, with a peak in the anterior hippocampus.

The spatial specificity of this source localisation is worth emphasising in light of our *a priori* region of interest, the hippocampus. This hypothesis arose from previous fMRI studies (Dalton et al., 2018; Hassabis et al., 2007b; Zeidman et al., 2015a,b) where robust activation of the anterior hippocampus has been observed during the mental construction of scene imagery. Due to the nature of the conjunction analysis, we cannot rule out that other nearby brain areas contributed to the process of mentally constructing scene imagery. However, we can conclude that the anterior hippocampus displayed the most consistent effect within the medial temporal lobe in the sample studied. A subsequent bootstrapped conjunction analysis confirmed a stable cluster of activation concentrated in the anterior hippocampus.

In the OP-MEG experiment we also observed power changes in posterior brain regions, namely the occipital cortex, calcarine sulcus, precuneus, cuneus and parietal cortex, as well as the ventromedial prefrontal cortex. In concert with the hippocampus, these areas constitute the “construction network” of the brain, which has been widely associated with imagination, episodic memory, future thinking and navigation (Spreng et al., 2009). The successful recovery of this core network from the limited (20–30) array of OP-MEG sensors used here, just 10% of the number of SQUID-MEG sensors, demonstrates not only their sensitivity but their wide range of applications for future research. Given that the accuracy of MEG source localisation increases with the number of available sensors (Vrba et al., 2004), we would expect further improvement in the spatial precision of OP-MEG with a larger number of sensors. The observed changes in theta power represented a decrease below the baseline counting condition. This finding adds to a growing body of literature which shows that successful memory processing in the hippocampus is associated with a concomitant decrease in theta power (Lega et al., 2012, 2017; Matsumoto et al., 2013; Sederberg et al., 2007).

Scanning the three OP-MEG participants in a SQUID-MEG scanner while they performed the same task, and subsequently applying the same analysis pipeline, afforded us the opportunity to compare the two technologies. In both OP-MEG and SQUID-MEG activation of the anterior medial temporal lobe was observed, with the peak of activation in this region consistently located in the hippocampus. Notably, while right-sided hippocampal theta changes were observed in OP-MEG, SQUID-MEG revealed left-sided hippocampal activation. In this respect, our findings align with the fMRI literature whereby studies involving mental scene construction have reported left (Addis et al., 2007; Howard et al., 2011), right (Addis et al., 2011; Lee et al., 2013) or bilateral (Hassabis et al., 2007b; McCormick et al., 2015; Zeidman et al., 2015a) hippocampal engagement. The factors which influence the extent to which hippocampal activity is lateralised during the generation and use of scene imagery remain to be specified. Other differences emerged between the two modalities at the group level, with OP-MEG more sensitive to power changes in posterior visual areas, while SQUID-MEG revealed strong left frontal activation during scene imagery. These variations may be attributable to the OP-MEG sensor placement in this study, which was optimised for detecting activity in the medial temporal lobe. Future OP-MEG studies using larger sensor arrays will yield more comprehensive coverage and thereby facilitate a better whole brain comparison with conventional SQUID-MEG.

An obvious advantage in the use of OP-MEG relative to SQUID-MEG is that of participant mobility. SQUID-MEG sensors remain in a fixed location within the scanner rather than in a fixed position relative to the participant. This means that any movement by the participant can compromise the accuracy of source reconstruction (Medvedovsky et al., 2007). While compensation can be made for a limited amount of movement (Uutela et al., 2001), the requirement to limit a participant’s movement also constrains the scientific investigations that can be conducted. The ability to perform brain imaging while participants are unconstrained will have a transformative impact on the study of cognitive processes in which movement is an integral part. The neural correlates of navigation, autobiographical memory, social interaction and motor skill learning, for example, can now be interrogated with greatly enhanced ecological validity which is precluded in SQUID-MEG and fMRI.

It should be noted, however, that the capacity to record brain activity in moving participants is not novel. For example, high-density EEG can be performed while participants are unconstrained. However, artefacts inherent to EEG recordings, such as muscle contraction, sweating, electrode, eye and tongue movement, respiration and heartbeat are exacerbated during active movement (Thompson et al., 2008). These complications are circumvented in the study of sports performance (Thompson et al., 2008), or real-world memory formation (Griffiths et al., 2016) by restricting the analysis of brain activity to stationary periods. Other solutions include methods to characterise and remove movement-related artefacts, which are continually being optimised (Stone et al., 2018). These approaches have allowed for the study of sensor and source-space neural correlates of basic (Ball et al., 2008) and expressive (Cruz-Garza et al., 2014) dance movements, as well as ambulation in both the real world (Seeber et al., 2014), and while navigating in immersive virtual reality (Liang et al., 2018), and in assessing how brain activity is affected by the perturbation of sensory input (Malcolm et al., 2018). Other studies have successfully recovered the neural evoked responses to cognitive tasks in both indoor and outdoor environments while participants concurrently engaged in active movement, such as arm extension (Jungnickel and Gramann, 2016), walking (Debener et al., 2012; Gramann et al., 2010), running (Gwin et al., 2010) and even cycling (Zink et al., 2016).

Therefore, are there any distinct advantages of using OP-MEG over EEG in moving participants? It remains a controversial issue whether residual artefacts from movement still pollute the EEG signal and lead to spurious results (Castermans et al., 2014; Nathan and Contreras-Vidal, 2016), and it has been demonstrated that some movement artefact may be erroneously localised to within the brain with EEG (Snyder et al., 2015). Muscle artefact in particular can obscure brain activity in the EEG recording of a moving participant (Claus et al., 2012; Muthukumaraswamy, 2013). By contrast, the muscle artefacts present in OP-MEG are lower than those in EEG by a factor of ten (Boto et al., 2018). Another clear benefit of using an MEG system is the relative insensitivity of the forward model to the effects of volume conduction which provides enhanced spatial resolution relative to EEG (Baillet, 2017; Hämäläinen et al., 1993).

A related issue is the relative suitability of each method for detecting deep sources such as the hippocampus. In theory, EEG has greater depth sensitivity than MEG (Goldenholz et al., 2009), assuming one can accurately model the volume conduction. The ability of MEG to detect activity in the medial temporal lobe has been questioned (Mikuni et al., 1997; Shigeto et al., 2002). However, cell density is higher in the hippocampus than the neocortex (Attal et al., 2012), and the corresponding increase in neural current should compensate for its depth, yielding equivalent detectability to much of the neocortex (Attal and Schwartz, 2013). Recent evidence from combined intracranial electrode recordings and MEG supports this perspective, where spontaneous hippocampal theta oscillations (Dalal et al., 2013) and interictal spikes (Pizzo et al., 2019) recorded from hippocampal electrodes in epilepsy surgery patients were simultaneously recorded by MEG sensors. Furthermore, intracranial recordings in patients and source-reconstructed activity in controls showed comparable decreases in hippocampal theta power during successful memory encoding (Crespo-García et al., 2016). These findings add to converging evidence that the hippocampus can be successfully imaged using MEG (Pu et al., 2018; Ruzich et al., 2019).

Given the improvement in signal amplitude which OP-MEG affords, combined with the aforementioned resistance to volume conduction effects and muscle artefact, OP-MEG is a viable and, we would argue, preferable alternative to EEG for imaging the hippocampus, particularly in moving participants. Moreover, given that SQUID-MEG cannot adapt to the size of a participant’s head, the placement of children in order to achieve optimal sensitivity is challenging because of their smaller head size. While custom MEG systems exist for children (Roberts et al., 2014), wearable OP-MEG systems offer more adaptability and allow for the flexible placement of sensors in paediatric populations.

It should be noted that the conjunction analysis performed here is not subject to the same interpretation as typical neuroimaging group analyses. In such studies one uses a sample of participants to make an inference concerning the population from which they were sampled (Friston et al., 1999, 2005; Worsley and Friston, 2000). For example: “the average participant from this population displays hippocampal activation during this task”. However, the conjunction analysis deployed here rejects a very specific null hypothesis, namely, that none of the participants displayed activity in a given brain region. In this context, the null hypothesis could be formulated as “OP-MEG cannot detect a signal from the hippocampus in any participant”. We have rejected this null hypothesis, thereby concluding that the hippocampus was engaged by the task performed and that OP-MEG, as with SQUID-MEG, could detect the associated activity.

In summary, the results presented here represent a proof-of-concept that OP-MEG can detect engagement of the human hippocampus. Future research is required to optimise OP-MEG technology. For example, detection of hippocampal signals may be improved further by larger sensor arrays and alternate configurations of sensor positions. Our findings have potentially far-reaching implications for the study of naturalistic human cognition and its compromise in the context of brain injury or disease.

## Funding

This work was supported by a Wellcome Collaborative Award to G.R.B., M.J.B. and R.B. [grant numbers 203257/Z/16/Z, 203257/B/16/Z]; a Wellcome Principal Research Fellowship to E.A.M. [grant number 210567/Z/18/Z]; a Centre Award to the Wellcome Centre for Human Neuroimaging [grant number 203147/Z/16/Z].

## Declarations of interest

The Wellcome Collaborative Award includes a collaborative agreement with the OPM manufacturer QuSpin.
